# Trabecular Bone Score as a Reliable Measure of Lumbar Spine Bone Microarchitecture in Acromegalic Patients

**DOI:** 10.3390/jcm11216374

**Published:** 2022-10-28

**Authors:** Elena Nazzari, Andrea Casabella, Sabrina Paolino, Claudia Campana, Giuliana Corica, Federica Nista, Angelo Milioto, Alberto Tagliafico, Manuela Albertelli, Mara Boschetti, Marcello Bagnasco, Maurizio Cutolo, Diego Ferone, Federico Gatto

**Affiliations:** 1 Endocrinology and Diabetology, ASL2 Savonese, 17100 Liguria, Italy; 2 Research Laboratory and Academic Division of Clinical Rheumatology, Department of Internal Medicine, University of Genoa, IRCCS Ospedale Policlinico San Martino, 16132 Genoa, Italy; 3 Endocrinology Unit, Department of Internal Medicine and Center of Excellence for Biomedical Research, University of Genoa, 16132 Genoa, Italy; 4 Department of Health Sciences (DISSAL), University of Genoa, 16132 Genoa, Italy; 5 Department of Radiology, IRCCS Ospedale Policlinico San Martino, 16132 Genoa, Italy; 6 Endocrinology Unit, IRCCS Ospedale Policlinico San Martino, 16132 Genoa, Italy

**Keywords:** acromegaly, trabecular bone score, disease control, bone mineral density, vertebral fractures, bone markers

## Abstract

Although GH and IGF-1 excess has a controversial impact on bone mineral density (BMD), acromegalic patients display variable degrees of bone structure impairment. In this study, we aim to investigate the usefulness of trabecular bone score (TBS), compared to BMD, in identifying acromegalic patients with impaired lumbar spine trabecular microarchitecture. Forty-four acromegalic patients were investigated for disease control, metabolic and gonadal status, bone metabolism parameters, and the presence of vertebral fractures (VFs). Patients and matched healthy controls underwent BMD and TBS examination. Mean TBS values were lower in patients than in controls (*p* < 0.001), without significant differences in mean lumbar and femoral BMD. TBS values were significantly higher in controlled patients compared to the uncontrolled ones (*p* = 0.012). No significant differences were found in bone markers with respect to disease control. Mean TBS or lumbar BMD did not significantly differ in patients with or without VFs (prevalence 11.4%). TBS and BMD levels were lower in hypogonadal patients compared to the eugonadal ones (*p* = 0.030 and *p* < 0.001, respectively). In conclusion, TBS values are significantly lower in patients than in controls, confirming the presence of impaired lumbar spine trabecular bone in acromegaly. Both uncontrolled disease and hypogonadism contribute to TBS deterioration in acromegaly.

## 1. Introduction

Acromegaly has long been considered a cause of secondary osteoporosis, although bone mineral density (BMD) is not always reduced in acromegalic patients.

Growth hormone (GH) and insulin-like growth factor 1 (IGF-1) have a predominantly anabolic effect on the bone. However, their long-term excess seems to activate bone reabsorption mechanisms, and to increase bone turnover with a controversial final effect on BMD [1,2,3,4]. Currently available data from the literature report both normal, increased, and also reduced bone density in acromegalic patients, compared to the general population. Furthermore, in patients with acromegaly, other factors can affect bone metabolism, particularly the presence of hypogonadism (with or without hyperprolactinemia), a condition often associated with the disease [5,6,7,8,9,10]. The effect of GH excess on bone can vary in relation to the analyzed sites, with normal or reduced bone density at the lumbar spine (70% trabecular bone), and increased in the appendicular bone (90% cortical bone) [11,12]. The final result is bone tissue characterized by an altered trabecular structure, which is more prone to the risk of fractures.

Despite a number of studies reporting an increased incidence of vertebral fractures (VFs) in acromegalic patients, a significant correlation between BMD values and the risk of fractures has never been found [6,13,14,15,16,17,18,19,20]. 

In order to better evaluate the trabecular bone structure in acromegalic patients, different techniques have been used, including peripheral quantitative computed tomography (pQTC) on iliac crest biopsy, quantitative vertebral computed tomography (QCT), high-resolution peripheral quantitative computed tomography (HR-pQCT), cone-beam computed tomography, histomorphometry of bone biopsy, and trabecular bone score (TBS) [6,20,21,22,23,24,25]. Among the recently developed methods to study the trabecular bone microarchitecture, TBS measurement is particularly accurate in identifying post-menopausal women at high risk of bone frailty and fractures, as well as in monitoring the efficacy of medical therapies [26,27,28,29]. TBS is an indirect quantitative index that classifies the state of trabecular bone microarchitecture, and it is calculated jointly with the densitometric results. The advantages of TBS compared to the other proposed techniques mainly resides in the use of a non-invasive investigation, performed in the same place as the standard densitometric examination, without exposing the patient to additional radiations, with a (very) low burden on health care costs. The evaluation of TBS has been proposed, in association with dual-energy x-ray absorptiometry (DXA), for the study of bone quality in various endocrine disorders such as primary hyperaldosteronism, Cushing’s syndrome and subclinical hypercortisolism, primary hyperparathyroidism, GH deficiency, as well as diabetes mellitus, proving in some cases more reliable than BMD alone in identifying patients with a higher risk of fractures [30,31,32,33,34,35,36,37,38,39].

Currently, few studies have analyzed TBS in patients with acromegaly [23,24,40,41,42,43,44,45,46,47]. In most cases, the authors reported lower TBS values in acromegalic patients compared to healthy subjects. However, the impact of disease control on TBS values is still controversial, and studies mainly focused on the effects of medical treatment on bone microarchitecture in patients with long-term follow-up are still lacking. 

Therefore, the main aims of the present study are: (i) to evaluate BMD (lumbar and femoral) and lumbar TBS values in a cohort of acromegalic patients compared to a control group of healthy subjects; (ii) to investigate the impact of disease control on both BMD and TBS values; (iii) to assess the prevalence of VFs in acromegalic patients, correlating the presence of VFs with BMD and TBS values. Furthermore, we investigated the role of RANK/RANK-L/OPG and DKK-1/sclerostin systems in our cohort, trying to elucidate the correlation between bone metabolism serum markers and TBS values, disease activity, as well as the presence of VFs.

## 2. Patients and Methods

### 2.1. Study Design and Patients

An observational cohort study involving acromegalic patients with active follow-up at a single tertiary center for pituitary diseases (Endocrinology Unit, IRCCS Ospedale Policlinico San Martino, Genoa, Italy). Diagnosis of acromegaly was made based on clinical features, biochemical evidence of GH hypersecretion (lack of suppression of GH to <1 µg/L after a 2-h oral glucose tolerance test), IGF-1 levels above the age-adjusted upper limit of normality range (>1 xULN), and the presence of a pituitary adenoma at magnetic resonance imaging (MRI). 

Forty-four acromegalic patients (28 females, age range 31–75 years) were included in the study, and 44 healthy volunteers comparable for age, sex, and BMI (35 females, age range 27–77 years) were enrolled as a control group. Pregnancy status was excluded for women of childbearing age. Patients with diseases or clinical conditions possibly leading to osteoporosis (hyperthyroidism/thyrotoxicosis, hypercortisolism, primary or secondary hyperparathyroidism, chronic renal failure, malabsorption, bedridden), as well as current or previous therapies that can impact bone structure (glucocorticoids as anti-inflammatory/immunosuppressive therapy, GnRH analogues, immunosuppressive drugs, antiretrovirals, anticoagulants, anticonvulsants, pioglitazone) were excluded from the study. The presence of uncontrolled diabetes mellitus (HbA1c ≥ 8%), active malignant neoplasms, and previous traumas represented additional exclusion criteria. 

Detailed clinical information was collected for all patients. Particularly, the presence of familial history of osteoporosis, lifestyle, smoking habits, and alcohol intake, as well as the time from diagnosis of acromegaly was investigated. 

As for the control group, the presence of primary or secondary osteoporosis, as well as previous or ongoing osteoporosis treatments were carefully investigated in the clinical history, and they were considered as exclusion criteria. 

At the time of data collection (data censoring: time of DXA and TBS assessment), IGF-1 values were used to evaluate patient biochemical control. In detail, according to recent clinical studies and consensus statements, we considered as having an acceptable biochemical control (controlled patients) those study subjects with sex- and age-adjusted IGF-1 values < 1.2 the upper limit of normality (ULN), while patients with IGF-1 levels ≥ 1.2 xULN were defined as uncontrolled [48,49,50]. Time from diagnosis was defined as the timeframe from the diagnosis and the time of data collection, irrespective of disease control. 

As concerns the gonadal status, patients were considered hypogonadal in case of low total testosterone levels and associated symptoms (men), or low levels of estradiol accompanied by the absence of menstrual cycles (women). Women with menopause were included in the hypogonadal group, as well. 

The study was conducted in accordance with the recommendations of the Declaration of Helsinki and all patients gave written informed consent to use clinical data for research purposes.

### 2.2. Laboratory Methods

The following hormonal and metabolic parameters were investigated in all acromegalic patients at the time of DXA and TBS assessment: GH, IGF-1, fasting plasma glucose, glycated hemoglobin (HbA1c), thyroid stimulating hormone (TSH), free thyroxine (fT4), prolactin (PRL), testosterone (men), estradiol (women), sex-hormone binding globulin (SHBG), albumin, morning plasma cortisol, urinary free cortisol (UFC), parathyroid hormone (PTH), 25-hydroxyvitamin D [25(OH)D], 1,25-dihydroxyvitamin D [1,25(OH)2D], electrolytes, creatinine, bone ALP and osteocalcin. All the above-mentioned parameters were determined by the routine automatic methods in use at the Medicine Laboratory of our Institution (IRCCS Ospedale Policlinico San Martino, Genova, Italy). Additional biomarkers of bone function (osteoprotegerin (OPG), sclerostin, Dickkopf-related protein 1 (DKK-1), RANK-L) were tested in acromegalic patients, being assessed at the Research Laboratory of Clinical Rheumatology of our hospital (by use of Enzyme Immunoassays; Biomedica Medizinprodukte GmbH, Wien, Austria).

### 2.3. BMD, TBS, and Vertebral Fracture Assessment

All study participants (both patients and controls) underwent DXA to evaluate the bone quantity and bone quality using BMD and TBS, respectively. Densitometry values were detected at the lumbar spine (L1-L4), and at all femoral sites (neck, ward’s triangle, trochanter, total hip), and they were computed using the Lunar Prodigy Advance densitometer (GE Lunar, enCORE software GE Healthcare version 16, Madison, WI, USA). 

BMD values were expressed as grams per square centimeter (g/cm^2^ ± SD), and as T-score, a measure of the bone quantity of the study subject compared with a young adult of the same gender with peak bone mass. A T-score below −2.5 identifies osteoporosis. We also evaluated the Z-score, which establishes the amount of bone compared with people of the same age and gender group [51]. During the same DXA procedure, TBS was calculated at the lumbar spine, using the region of interest of the BMD measurement, by use of the iNsight software (Medimaps^®^). All acromegalic patients also underwent dorsal-lumbar spine x-ray morphometric analysis to detect vertebral fractures.

The results were correlated with biochemical control, bone metabolism parameters, and metabolic and gonadal status at the time of data censoring (namely, the time of DXA and TBS assessment).

### 2.4. Statistical Analysis 

SPSS 28.0 for Windows (SPSS, Chicago, IL, USA) was used for statistical analyses, while GraphPad Prism version 5.01 (GraphPad Software, San Diego, CA, USA) was used to draw figures. The Kolmogorov-Smirnov test was used to check the normality of the distribution of the continuous variables. Quantitative data are presented as mean ± standard deviation (SD), while categorical variables are presented as frequencies and percentages. Between-group comparisons were analyzed by the Student’s *t*-tests (or the Mann–Whitney test) and the one-way ANOVA test (or the Kruskal–Wallis test), where appropriate. Correlations were performed using Pearson’s *r* correlation coefficient or Spearman’s rho correlation coefficient for ranks, based on data distribution. The two-sided Fisher’s test or the Chi-square test was used to evaluate differences in cross-tables. Differences were taken to be statistically significant at *p* < 0.05.

## 3. Results

### 3.1. Patients Characteristics

Forty-four patients with acromegaly (28 females, 16 males) and 44 healthy controls (35 females, 9 males) were included in the study (F/M ratio between patients and controls: *p =* 0.155). Mean age was 54.2 (± 11.5) years for patients, and 51.3 (± 11.2) years for controls (*p =* 0.430). No significant differences were found in BMI values between patients and controls (26.96 ± 5.37 Kg/m^2^ vs. 25.17 ± 4.55 Kg/m^2^, *p =* 0.128).

General and clinical patient characteristics, including time from diagnosis, family history for osteoporosis, alcohol intake, and smoking habits, as well as treatment modalities and biochemical outcomes, are depicted in Table 1. In detail, osteoporosis was reported in the family history for five patients, while 10 study subjects reported smoking habits. Regarding alcohol consumption, 24 patients reported occasional intake, 6 moderate consumption (two alcoholic units/day for men and one alcoholic unit/day for women), and only 1 patient reported high alcohol consumption. 

At the time of DXA and TBS assessment, mean GH and mean absolute IGF-1 values were 2.25 ± 1.92 μg/L and 269.70 ± 201.18 μg/L, respectively. Mean sex- and age-adjusted IGF-1 levels were 1.14 ± 0.83 xULN. Biochemical control, defined as IGF-1 xULN < 1.2 xULN, was detected in 34 patients (77%, mean IGF-1 0.79 ± 0.20 xULN), while 10 patients were considered as uncontrolled (23%, mean IGF-1 2.34 ± 1.03 xULN). Twenty-one out of 44 patients (48%, 5 males and 16 females) had hypogonadism due to menopause or pituitary function impairment.

The mean hormone and biochemical parameters of the patient cohort are reported in Table 2. Overall, free thyroid hormones, morning plasma cortisol, and UFC were within the normal range in all patients, while only one study subject presented with mild hyperprolactinemia (44 µg/L).

As for 25(OH)D, 16/44 patients (36%) had values <20 ng/mL (mean 21.97 ± 7.72 ng/mL). However, calcium levels and PTH values were normal in all patients. As expected, 25(OH)D values directly correlated with both calcium (*r* = 0.307, *p =* 0.042) and 1,25(OH)2D levels (rho = 0.439, *p =* 0.003), while showing an inverse correlation with PTH (*r* = −0.367, *p =* 0.014). Furthermore, PTH levels were directly correlated with age (*r* = 0.398, *p =* 0.007) and osteocalcin levels (*r* = 0.352, *p =* 0.022).

### 3.2. TBS and BMD Values in Acromegalic Patients and Healthy Subjects

**Figure 1:**
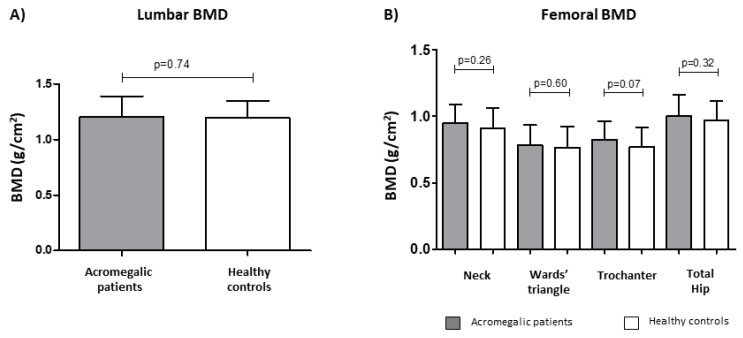
Mean lumbar (**A**) and femoral (**B**) bone mass density (BMD) values did not significantly differ in acromegalic patients (*n* = 44) compared to age- and sex-matched healthy controls (*n* = 44).

**Figure 2:**
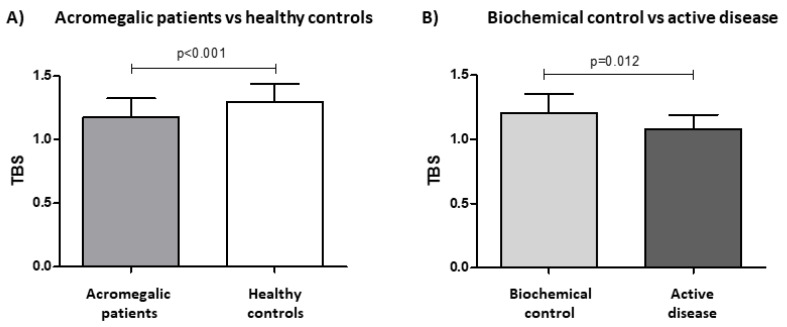
Mean trabecular bone score (TBS) values were significantly lower in acromegalic patients compared to age- and sex-matched healthy controls (**A**). Patients achieving biochemical control (IGF-1 <1.2 xULN) had higher TBS values compared to the uncontrolled ones (**B**).

Patients and healthy controls were not significantly different in BMD values, T-score, and Z-score at both the lumbar spine (L1-L4) and all femoral sites (Figure 1, values detailed in Table 3). Of note, mean TBS values were significantly lower in acromegalic patients compared to healthy controls (1.18 ± 0.15 vs. 1.29 ± 0.14, *p* < 0.001) (Figure 2A, Table 3). 

We observed a significant inverse correlation between TBS values and patients’ age (*r* = −0.44, *p =* 0.002), whereas sex, BMI, lifestyle, smoking habits, alcohol intake, familial history of osteoporosis, and time from diagnosis, did not significantly affect TBS. Interestingly, TBS in the control group was not significantly correlated with age (*r* = −0.22, *p =* 0.151), and, similarly to the observation in the patient group, sex and BMI did not affect TBS values, as well. 

Stratifying acromegalic patients based on IGF-1 levels, we found that controlled subjects (IGF-1 < 1.2 xULN) had higher TBS values compared to the uncontrolled ones (1.21 ± 0.15 vs. 1.08 ± 0.12, *p =* 0.013). In this context, we observed a trend for an inverse correlation (although not statistically significant) between TBS values and sex- and age-adjusted IGF-1 levels (rho= −0.277, *p =* 0.068). On the contrary, GH and absolute IGF-1 values did not correlate with TBS (rho = 0.03, *p =* 0.854 and rho= −0.191, *p =* 0.213, respectively).

When considering the other hormonal and biochemical parameters evaluated in acromegalic patients (see Laboratory Methods), we found a significant inverse correlation between fasting plasma glucose levels and TBS values (*r* = −0.408, *p =* 0.006). On the other hand, HbA1c, thyroid function, cortisol levels, PRL, 25(OH)D, 1,2(OH)2D, Ca, P, PTH, bone ALP, and osteocalcin did not correlate with TBS.

Mean TBS values were significantly lower in hypogonadal patients than in the eugonadal ones (1.13 ± 0.12 vs. 1.22 ± 0.16, *p =* 0.016). Similarly, BMD values at all sites were significantly lower in hypogonadal compared to eugonadal patients (lumbar: 1.10 ± 0.17 vs. 1.30 ± 0.14, *p* < 0.001; femoral neck: 0.88 ± 0.12 vs. 1.01 ± 0.14, *p* < 0.001; ward’s triangle: 0.70 ± 0.11 vs. 0.86 ± 0.15, *p* < 0.001; trochanter: 0.74 ± 0.11 vs. 0.90 ± 0.12, *p* < 0.001; total hip: 0.91 ± 0.12 vs. 1.09 ± 0.13, *p* < 0.001). A significant direct correlation was found between estradiol levels and TBS in females (*r* = 0.668, *p =* 0.005), whereas it was not detected between total testosterone levels and TBS values in men (*r* = 0.352, *p =* 0.181).

Of note, the prevalence of hypogonadism was higher among patients with active disease (8/10, 80%), compared to the controlled ones (13/34, 38%; Fisher’s test *p =* 0.031). Within controlled acromegalic patients, we did not observe any statistically significant difference in TBS values between hypogonadal and eugonadal acromegalic patients (1.16 ± 0.11 vs. 1.24 ± 0.16, *p =* 0.131). 

At univariate linear regression analysis, both uncontrolled disease (adjusted R^2^: 0.117, B: −0.130, β: −0.371, *p =* 0.013) and hypogonadism (adjusted R^2:^ 0.086, B: −0.096, β: −0.327, *p =* 0.030) were significant negative predictors of TBS values. When the concomitant presence of uncontrolled disease and hypogonadism was evaluated using a multivariate regression model, we found an adjusted R^2^ value of 0.142. Of note, age was another significant predictor of TBS value, both in univariate and multivariate analysis. Interestingly, when considering uncontrolled disease, hypogonadism, and age in the multivariate regression model, the adjusted R^2^ value raised to 0.256, with uncontrolled disease and age being still independent significant predictors of TBS (Table 4). 

### 3.3. RANK-L/Osteoprotegerin and DKK-1/Sclerostin System in Acromegalic Patients

We found that biochemically controlled patients had slightly higher RANK-L levels (357.36 ± 297.68 vs. 262.33 ± 394.14 pg/mL, *p =* 0.092) and RANK-L/OPG ratio (18.03 ± 14.66 vs. 10.44 ± 14.49, *p =* 0.060), compared to the uncontrolled ones, although these differences were not statistically significant. No differences were found in OPG (16.43 ± 9.64 vs. 20.04 ± 9.77 pg/mL, *p =* 0.374), DKK-1 (1841.92 ± 786.7 vs. 1329.67 ± 1056.98 pg/mL, *p =* 0.102) and sclerostin (59.60 ± 33.73 vs. 51.39 ± 37.55 pg/mL, *p =* 0.529) levels between controlled and uncontrolled patients. Of note, all the bone markers investigated showed a large variability among patients, irrespective of disease control, as demonstrated by the high standard deviation values reported above.

No statistically significant correlations were found between GH, IGF-1 levels, and bone markers, except for an inverse correlation between sclerostin and absolute IGF-1 values (rho= −0.343, *p =* 0.024). 

TBS values in acromegalic patients did not correlate with RANK-L, OPG, DKK1, and sclerostin levels. Overall, bone markers did not correlate with patient BMD, except for a significant direct correlation observed between RANKL/OPG ratio and trochanteric BMD values (rho = 0.340, *p =* 0.037).

### 3.4. TBS, BMD, Bone Markers, and Vertebral Fractures

In our cohort, the prevalence of silent VFs (assessed by vertebral morphometry) was relatively low (5 out of 44 patients, 11.4%). No significant differences were found in mean lumbar BMD (*p =* 0.565) and TBS values (*p =* 0.858) in patients with VFs compared with those without fractures. 

General patient characteristics, time from diagnosis, as well as GH, IGF-1 values, and bone markers were not significantly associated with the presence of VFs. 

## 4. Discussion

In the present study, we found that TBS values were lower in acromegalic patients compared to age, BMI, and sex-matched healthy controls, while no significant differences were observed in BMD values between the two groups, both at lumbar and femoral sites. These data are in line with the results reported by other authors, although some studies did not find significant differences in TBS values between patients and matched controls [23,41,42,43,45,46,47]. Furthermore, we observed that both BMD and TBS values were significantly lower in hypogonadal patients compared to the eugonadal ones, thus confirming the pivotal role of gonadal status on both bone density and quality in acromegaly [6,40]. 

In line with previous findings, we observed a significant inverse correlation between TBS values and age in acromegalic patients, although this correlation was not found in the control group [23,37,43,46].

We observed a slight (not statistically significant) trend for an inverse correlation between TBS and sex- and age-adjusted IGF-1 values, while both GH and absolute IGF-1 levels were not major determinants of TBS values. However, stratifying our patients based on biochemical control, we found that controlled subjects had higher TBS values compared to those with active disease. Of note, the vast majority of our patients achieved biochemical control following medical therapy, and at the time of TBS evaluation had a long disease history (median follow-up 13.98 ± 6.20 years).

This finding is of particular interest, since the effect of disease control on bone metabolism in acromegaly remains controversial. Indeed, Calatayud and colleagues reported higher TBS values in patients who underwent post-surgical remission [43], while a previous report from Godang K et al. described a reduction in TBS values one year after surgery, although associated with an increase in BMD levels [24]. Recently, Sala and colleagues failed to demonstrate significant changes in both TBS and BMD values in a prospective study evaluating 18 acromegalic patients at diagnosis and 12 months after achieving cured/controlled disease (66.7% of the patients were treated with somatostatin receptor ligands) [44].

As concerns the role of RANK/RANK-L/OPG and DKK-1/sclerostin systems in acromegaly, many aspects remain to be clarified. We did not find any significant difference in bone markers between controlled and uncontrolled patients. While no significant correlations were found between GH and IGF-1 levels and OPG, DKK-1, and RANK-L, we observed an inverse correlation between IGF-1 and sclerostin values. Interestingly, another study showed an inverse correlation between sclerostin and GH levels, suggesting that the observed decrease in Wnt antagonists’ levels (such as sclerostin) could represent a compensatory mechanism to counteract the increased bone frailty in active acromegaly [52]. 

However, another study showed a positive correlation between sclerostin, GH, and IGF-1 values [53], while Uygur and colleagues recently reported a lack of correlation between sclerostin with both GH and IGF-1 values [54]. Therefore, the significance of sclerostin levels in acromegaly is still debated, and the impact of disease control on sclerostin is still unknown. 

The role of other bone markers in both active and controlled acromegaly is largely debated. In contrast with our results, Ozer and coworkers reported an inverse correlation between OPG and IGF-1 levels [55], while Constantin and colleagues did not find any correlation between GH and IGF-1 with both OPG and RANK-L [56]. High DKK-1 levels have been already reported in patients with acromegaly, and some studies describe an increase of this marker in patients with GH deficiency following GH replacement therapy [40,57]. In this light, Belaya and colleagues recently reported that GH excess results in an increased expression of DKK-1 [58].

The pathogenesis of increased bone resorption in acromegaly remains unclear. Osteoclasts express IGF-1 and IGF-1 receptors, therefore the GH/IGF-1 system can stimulate the production of cytokines involved in osteoclast regulation. Moreover, the complex interaction between osteoclasts and adipocytes observed in acromegaly may also play a role [40]. Studies on animal models and GH-deficient patients suggested that the RANK-L/OPG system might mediate the effects of IGF-1 on osteoclasts [59,60]. In this light, we would expect a decrease in the RANK-L/OPG ratio after acromegaly treatment. However, we found that controlled patients had slightly higher RANK-L and RANK-L/OPG ratios compared to patients with active disease, while no difference was found in OPG levels. Therefore, our results, together with previous data reported by other authors, did not confirm this hypothesis [56]. A possible explanation could be that plasma RANK-L and OPG levels may not reflect local cytokine production at the tissue level.

Our data confirm the presence of silent VFs in acromegalic patients. In our cohort, the prevalence of VFs, assessed by morphometric examination, was 11.4%, similar to that reported by Madeira and colleagues, but lower compared to other studies [13,14,15,16,17,18,19,20,23,41,61]. Different methods have been used to identify VFs in different studies, performing a radiographic examination of the spine rather than a morphometric examination in most cases. These differences can, at least partially, explain the heterogeneity observed in the reported prevalence of VFs. We did not find significant differences in TBS values and bone markers between patients with or without VFs. However, these data need to be carefully handled, due to the low prevalence of VFs found in our cohort.

Overall, the main strength of our study is the comprehensive evaluation of BMD, TBS, VFs, disease control, biochemical and hormonal parameters, as well as specific bone markers in a well-characterized cohort of acromegalic patients followed-up at a tertiary center for pituitary diseases. 

The main limitations are represented by the relatively limited number of patients (although carefully selected and characterized), and the absolute low number of subjects with VFs (only five subjects).

Due to the complex mechanisms regulating the cross-talk between bone density, bone structure, bone markers, and the GH/IGF-1 system, our results need to be investigated possibly in larger cohorts [62].

In conclusion, we have confirmed that acromegalic patients have significantly lower TBS values than matched healthy subjects, without significant differences in BMD values and scores. These results highlight the impairment of trabecular bone in acromegalic patients, underling how TBS and BMD provide different information about the bone status. The evaluation of TBS is useful in identifying acromegalic patients with deterioration of trabecular structure at the lumbar spine and, therefore, possibly exposed to a higher risk of VFs. TBS evaluation can be carried out in the same session as standard densitometry, saving time, and money, and preventing patients to further exposure to ionizing radiation compared to other methods used to study trabecular bone structure. In this context, particular attention should be given to elderly patients and those with concomitant impairment of gonadal function.

## Figures and Tables

**Table 1:** General, clinical characteristics and hormone values of the acromegalic patients included in the study. Available data about healthy controls are reported, as well.

	Patients (*n* = 44)	Healthy Controls (*n* = 44)	*p*-Value
Sex (F; *n*, %)	28 (63.6)	35 (79.5)	0.155
Age (mean ± SD; years)	54.2 ± 11.5	51.3 ± 11.2	0.430
BMI (mean ± SD; Kg/m^2^)	26.96 ± 5.37	25.17 ± 4.55	0.128
Hypogonadism/menopause (*n*, %)	21 (48)	16 (36)	0.388
Familial history of osteoporosis (*n*, %)	5 (11.4)		
Smoking habits (*n*, %)	10 (22.7)		
Alcohol intake ^a^ (*n*, %) -no consumption -sporadic -moderate -high	13 (29.5) 24 (54.5) 6 (13.6) 1 (2.4)		
Lifestyle (*n*, %) -sedentary -mild activity -moderate activity -intense activity	13 (29.5) 20 (45.5) 9 (20.5) 2 (4.5)		
Time from diagnosis (mean ± SD; years)	13.98 ± 6.20		
**Treatment modalities**			
Neurosurgery	29 (65.9)		
Radiotherapy	2 (4.5)		
**Medical therapy**			
Fg-SRL	26 (59.1)		
Fg-SRL + CAB	4 (9.1)		
Fg-SRL+ PEG	5 (11.4)		
Fg-SRL + CAB + PEG	2 (4.5)		
**Biochemical values** (last follow-up)			
GH (mean ± SD; µg/L)	2.25 ± 1.92		
IGF-1 absolute (mean ± SD; µg/L)	269.7 ± 201.18		
IGF-1 xULN (mean ± SD)	1.14 ± 0.82		
Biochemical control (IGF-1 < 1.2 xULN; *n*,%)	34 (77.3)		

**Legend:** F, females; SD, standar deviation; BMI, body mass index; fg-SRL, first-generation somatostatin receptor ligand; PEG, pegvisomant; CAB, cabergoline; GH, growth hormone; IGF-1, insulin-like growth factor 1; ULN, upper limit of normality range. ^a^ alcohol intake: sporadic, <2 alcoholic units/day for men and <1 alcoholic unit/day for women; mild consumption, 2 alcoholic units/day for men and 1 alcoholic unit/day for women; high consumption: >2 alcoholic units/day for men and >1 alcoholic unit/day for women.

**Table 2:** Main hormonal and biochemical parameters investigated in acromegalic patients.

Measures	Values (Mean ± SD)	Normal Ranges
Fasting plasma glucose (mg/dL)	93.43 ± 16.81	65–110
HbA1c (%)	5.92 ± 0.48	4.3–5.8
PRL (µg/L)	8.50 ± 7.95	M: 2.64–13.13 F: 3.34–26.72 ^1^; 2.74–19.64 ^2^
TSH (mIU/L)	1.41 ± 0.83	0.27–4.20
fT4 (ng/L)	12.17 ± 1.99	9.3–17.0
Morning plasma cortisol (µg/dL)	11.28 ± 2.85	3.7–19.4
UFC (µg/24 h)	37.50 ± 20.76	4.3–176.0
25(OH)D (ng/mL)	21.97± 7.72	6.0–46.0
1,25(OH)2D (pmol/L)	148.01 ± 69.66	43–168
PTH (ng/L)	25.58 ± 8.25	6.5–36.8
Ca (mg/dL)	9.61 ± 0.36	8.5–11.0
P (mg/dL)	3.25 ± 0.58	2.5–4.5
Mg (mg/dL)	2.04 ± 0.21	1.9–2.5
Osteocalcin (µg/L)	16.90 ± 6.68	M: 12.0–52.1 F: 6.5–42.3 ^1^; 5.4–59.1 ^2^
Bone ALP (µg/L)	8.15 ± 3.51	M: 8–16.6 F: 5.8–11.6 ^1^; 8.5–17.9 ^2^

**Legend:** HbA1c, glycated hemoglobin; PRL, prolactin; M, males; F, females; TSH, thyroid stimulating hormone; fT4, free thyroxine; UFC, urinary free cortisol; PTH, parathyroid hormone; 25(OH)D, 25-hydroxyvitamin D; 1,25(OH)2D, 1,25-dihydroxyvitamin D; Ca, calcium, P, phosphorus; Mg, magnesium; ALP, alkaline phosphatase. ^1^ normal ranges for pre-menopausal females; ^2^ normal ranges for post-menopausal females.

**Table 3:** Mean TBS (lumbar), BMD, T-score, and Z-score (all sites) in acromegalic patients and matched healthy controls.

Data	Patients (*n* = 44)	Healthy Controls (*n* = 44)	
**TBS**	1.18 ± 0.15	1.29 ± 0.14	***p* < 0.001**
**Lumbar BMD (L1–L4)**			
BMD (g/cm^2^)	1.20 ± 0.19	1.19 ± 0.16	*p =* 0.74
T-score	0.09 ± 1.52	−0.07 ± 1.10	*p =* 0.61
Z-score	0.58 ± 1.25	0.56 ± 1.11	*p =* 0.93
**Femoral BMD**			
Neck BMD (g/cm^2^)	0.95 ± 0.14	0.91 ± 0.15	*p =* 0.13
Neck T-score	−0.52 ± 1.08	−0.73 ± 1.12	*p =* 0.37
Neck Z-score	0.09 ± 0.82	−0.11 ±1.07	*p =* 0.32
Ward’s triangle BMD (g/cm^2^)	0.78 ± 0.15	0.77 ± 0.16	*p =* 0.60
Ward’s triangle T-score	−1.12 ± 1.09	−1.11 ± 1.21	*p =* 0.98
Ward’s triangle Z-score	−0.08 ± 0.80	−0.17 ± 1.05	*p =* 0.65
Trochanter BMD (g/cm^2^)	0.82 ± 0.14	0.77 ± 0.15	*p =* 0.07
Trochanter T-score	−0.12 ±1.13	−0.45 ± 1.12	*p =* 0.17
Trochanter Z-score	0.06 ± 0.99	−0.18 ± 1.03	*p =* 0.26
Total hip BMD (g/cm^2^)	1.00 ± 0.16	0.97 ± 0.15	*p =* 0.17
Total hip T-score	−0.22 ± 1.17	−0.39 ± 1.04	*p =* 0.48
Total hip Z-score	0.16 ± 0.90	0.03 ± 0.99	*p =* 0.54

**Legend to Table 3:** TBS, trabecular bone score; BMD, bone mass density. Bold text indicates a statistically significant difference with a p-value less than 0.05.

**Table 4:** Univariate and multivariate analysis evaluating the main predictors of TBS values in acromegalic patients.

	Univariate Analysis				Multivariate Analysis			
Variable	Adjusted R^2^	B	β	*p*-Value	Adjusted R^2^	B	β	*p* Value
Uncontrolled disease	0.117	−0.130	−0.371	**0.013**	-	−0.124	−0.353	**0.017**
Hypogonadism	0.086	−0.096	−0.327	**0.030**	-	0.029	0.098	0.600
Age	0.179	−0.006	−0.445	**0.002**	-	−0.006	−0.469	**0.010**
All variables					0.256			

**Legend to Table 4:** B, unstandardized B; β, standardized coefficient β. Bold text indicates statistical significance (*p*-value less than 0.05).

## Data Availability

The data presented in this study are available on request from the corresponding author.
